# Learnings from Patient-Report Workshop on Disease Progression in Myotonic Dystrophy

**DOI:** 10.1007/s43441-019-00017-1

**Published:** 2019-12-09

**Authors:** Molly White

**Affiliations:** Myotonic Dystrophy Foundation, 1004A O’Reilly Avenue, San Francisco, CA 94129 USA

**Keywords:** Myotonic dystrophy, Progression, PFDD, MDF

## Abstract

For researchers, pharmaceutical companies, and regulatory agencies involved in the development of treatments that slow or stop disease progression, the highly variable and unpredictable progression of symptoms in myotonic dystrophy (DM) makes it difficult to assess whether an intervention provides clinically-meaningful benefit on disease progression for patients. In evaluating the effectiveness of treatments, drug developers and regulators need to understand what triggers progression, since the presence or absence of triggers may influence symptoms in ways that could complicate the assessment of progression. To better understand disease burden and what people living with DM view as meaningful benefits, Myotonic (formerly Myotonic Dystrophy Foundation) convened a meeting based on the US Food and Drug Administration’s Patient-focused Drug Development initiative, at the Myotonic Annual Conference in September 2018. Over 300 people living with DM met to share with regulators and drug developers how disease progression has affected their lives and what they would consider to be the most beneficial treatment effects of progression-targeting therapies. A panel of five adults with DM (three with DM1 and two with DM2) was asked to describe when disease symptoms took a turn for the worse, what seemed to trigger disease progression, what treatment approaches have proven useful, and what characteristics panel participants most desire in a new treatment. Audience members were then invited to share their experiences. Specific triggers of progression vary from person to person, even within a single family. Among the triggers most frequently cited by meeting participants were physical and emotional stress, lack of sleep, illness, injury, surgery, pregnancy, overdoing it, and lack of activity. Illness in general was said to trigger symptoms. Slowing or stopping disease progression is viewed by people with DM as the most important objective of therapy.

## Introduction

Myotonic dystrophy types 1 and 2 (DM1 and DM2) are the primary genotypes of a progressive, multi-systemic genetic disorder and the most common type of adult-onset muscular dystrophy, with a population-based prevalence in DM1 of 1:2300 [1] and a prevalence in DM2 that may be higher in some populations. For the hundreds of thousands of people affected by DM, the unpredictable course of the disease makes planning for the future difficult and can lead to a decline in quality of life, as well as a sense of hopelessness and despair [2].

For researchers, pharmaceutical companies, and regulatory agencies seeking treatments that slow or stop disease progression, the highly variable and unpredictable progression of symptoms in DM makes it difficult to assess whether an intervention provides clinically-meaningful benefit on disease progression for patients.

To better understand disease burden and what people living with DM view as meaningful benefits, Myotonic convened a series of meetings designed to provide patients and families the opportunity to provide their perspectives to regulators and drug developers.

The first of these meetings, in September 2016, followed the rubric outlined in the Food and Drug Administration’s (FDA’s) Patient-focused Drug Development (PFDD) Initiative, which was launched in 2012 to capture patient and caregiver perspectives on diseases with unmet medical needs [3].

The FDA sponsored and conducted 20 PFDD meetings across a wide range of disease conditions, and then expanded the program by opening it to external groups such as Myotonic. The Myotonic PFDD meeting was the first externally-led meeting, and resulted in a Voice of the Patient report that was presented to the FDA in May 2017 [4]. Subsequently, Myotonic held a similarly-formatted and conducted meeting in 2017 where patients and caregivers discussed the central nervous system (CNS) manifestations of DM, as well as clinically-meaningful benefit for CNS-targeting therapies; and in 2018, the organization held a similar meeting for this community to discuss disease progression.

## Overview of DM

There are two major types of myotonic dystrophy (DM): type 1 (DM1) and type 2 (DM2), which are caused by mutations in two different genes. DM1 symptoms typically develop in adulthood, although childhood-onset, congenital and late-onset forms also exist. Prominent features of DM1 include muscle weakness, atrophy, and wasting, which can lead to impairments of mobility and manual dexterity, as well as respiratory impairments and heart defects. In addition, impaired smooth muscle function in the gastrointestinal system, uterus, gall bladder, and circulatory systems may cause a range of problems from diarrhea or constipation to problems with childbirth, gallstones, and low blood pressure.

The eyes may be affected by premature cataracts and weakness of the eyelid muscles. The central nervous and endocrine systems may also be affected, leading to cognitive impairment, disorders of sleep regulation, psychological problems, depression, an increased prevalence of diabetes, and infertility.

Newborns with the congenital form of DM1 typically show the most severe symptoms, including developmental delay, severe muscle weakness, and low muscle tone [5]. Onset in childhood is typically associated with cognitive impairment and behavioral abnormalities.

DM2 also affects multiple systems, especially skeletal muscles, heart, and brain, but it is typically considered a milder genotype than DM1. Symptom onset typically occurs in the 40s. Muscle pain is more severe in DM2 than in DM1, and CNS symptoms, including anxiety, depression, social withdrawal, and cognitive impairment, are common.

The DM1 and DM2 genetic mutations are caused by triplet repeat DNA segments, with the number of copies roughly correlating to disease severity in DM1 [6]. In DM2, the number of repeats does not strongly correlate with disease severity or age of onset. Due to anticipation, the DM1 repeat number increases over the course of a person’s life and when transmitted to subsequent generations, which leads to high variability in clinical expression even within a single family. Typically the disease becomes progressively more severe and with an earlier onset as it is passed on from one generation to the next. In DM2, anticipation is less pronounced.

## Development of Clinically-Meaningful Treatments

To develop effective treatments that will slow or stop progression of DM, investigators need to develop measures that reliably capture disease changes over time. To date little is known about myotonic dystrophy disease progression, other than the fact that it is slow and highly variable from patient to patient [7]. Functional measures such as muscle strength and respiratory capacity are commonly used in DM clinical studies. However, in recent years the U.S. Food and Drug Administration (FDA) has increasingly sought to include patients’ experiences, perspectives, needs, and priorities with respect to disease burden and clinically-meaningful benefit in drug development and evaluation processes. As experts in what it is like to live with their condition, the FDA believes patients are uniquely positioned to inform the understanding of the therapeutic context for drug development and evaluation.

In evaluating the effectiveness of treatments, drug developers and regulators also need to understand what triggers progression, since the presence or absence of triggers may influence symptoms in ways that could complicate the assessment of progression. At the Myotonic Annual Conference held in Nashville, TN in September 2018, over 300 patients and caregivers attending the conference met in general session via another PFDD-type workshop to share how disease progression has affected their lives and what they would consider to be the most beneficial treatment effects of progression-targeting therapies. Additional participation was conducted via livestream, and those attendees were encouraged to provide email-based responses to the workshop questions after the event. Two were received and incorporated into this report. Conference attendees were fairly evenly distributed across gender, and skewed slightly higher in family members and caregivers (58%) than in affected patients (42%), although some caregivers are also living with the disease, but identified primarily in the caregiver category.

### Voices of People with DM and Their Family Members

Session moderator Dr. Charles Thornton opened the 1–1/2-h session by describing the workshop goals, which included capturing patient and caregiver perspectives on the experience of disease progression or change over time, including progression triggers and strategies for managing progression and disease change. The workshop is part of Myotonic’s broader commitment to informing drug developers, regulators, and others about myotonic dystrophy symptomatology and disease burden, and patient priorities for therapy development. Dr. Thornton also provide an overview of the workshop structure, which began with a panel of five adults with DM (three with DM1 and two with DM2) reading prepared answers to queries developed via the PFDD model. These included: when their disease took a turn for the worse, what seemed to trigger disease progression, what treatment approaches had proven useful, and what characteristics they most desire in a new treatment. Dr. Thornton then moderated audience participation, inviting conference attendees to share their names, disease phenotype, and the age at which they began to experience significant disease change. The comments from panelists and audience members highlighted key issues that patients consider of primary importance in the development of more effective interventions as well as factors that may complicate the design of clinical trials, especially the substantial heterogeneity in symptoms, triggers of worsening disease, patterns of disease progression, and effectiveness of treatments.

#### Heterogeneity of Symptoms

The nature of symptoms varies substantially among people with DM. Fatigue and excessive daytime sleepiness were cited frequently as the most debilitating symptoms for those with both DM1 and DM2. For one man with DM1, however, cardiac symptoms including atrial flutter, atrial fibrillation, and heart-block arrhythmias have proven most debilitating [8].

Other symptoms noted by some individuals included cataracts, muscle weakness, myotonia, choking, slurring words, and cognitive decline. One man with DM1 described how executive function impairment has led to difficulty in planning, organizing and making decisions, problems that are exacerbated by apathy, lack of motivation, and constant fatigue [8]. A woman with DM2 described herself as “intellectually falling apart,” which she also said was exacerbated by fatigue and consumption of pain medications [8]. Another woman with DM2 described painful sensitivity to cold and sensitivity to anesthesia as major symptoms [8].

#### Variability of Triggering Events

Specific triggers of progression vary from person to person, even within a single family. Among the triggers most frequently cited by meeting participants were physical and emotional stress, lack of sleep, illness, injury, surgery, pregnancy, overdoing it, and lack of activity. Illness in general was said to trigger symptoms. One woman with DM2 added that illnesses linger for a long time [8]. Another woman said she became severely anemic at age 14 and never regained her stamina and endurance and added that a diagnosis of DM1 more than a decade later came as something of a relief in that it gave her an explanation and confirmed that she was not simply lazy [8]. A woman caring for her husband with DM1 said that he had fallen and broken an elbow before being diagnosed, and within 2 months he developed cataracts. He fell again and broke an ankle, which took 2 years to heal, during which time he experienced dramatically accelerated symptoms of fatigue, respiratory problems and heart-block arrhythmias [8]. Similarly, a woman with DM2 said a fall and broken hip had triggered worsening symptoms for her father but added that emotional stress triggers symptoms for other family members [8].

#### Variable Effectiveness of Interventions

Given the lack of effective treatments or cures for DM, people with the disease describe a wide range of management strategies. Strategies that have showed effectiveness in some meeting participants include drugs to help with excessive daytime sleepiness (modafinil, Provigil, Nuvigil, Adderall), myotonia (mexiletine), pain (gabapentin, naproxen, cannabidiol), depression and anxiety [8]. Physical therapy, aqua therapy, exercise, yoga, and Pilates were mentioned as helpful, as well as other lifestyle changes, including diet, nutritional supplements, and meditation. Some people said they benefited simply from adjusting their standards, paying closer attention to how they are feeling, and taking a break when needed [8].

Exercise was mentioned by many meeting participants as a deterrent to disease progression but also as a potential accelerant when done to excess. For example, a man who described himself as “an avid runner” was diagnosed with late-onset DM1 after experiencing fatigue and muscle atrophy. When he stopped running, the disease progressed more rapidly [8]. Another man with DM1 said he was running about 100 miles [8]. A mother with a daughter with childhood-onset DM1 wondered how much exercise is too much. She said her daughter dances 14 h a week and gets physical therapy. She has found dancing to be beneficial because it allows her to exercise different muscles and adjust her activity to match her level of endurance [8]. A man with DM1 said, “You need to stay as active as you can, but don’t overdo it” [8].

The availability of new reproductive technologies, such as preimplantation diagnosis, was also mentioned by a woman with DM1 as an intervention that can provide meaningful benefit by alleviating the burden of passing on the disease to children.

#### Agreement on What Would Be a Meaningful Treatment

Meeting participants agreed that a meaningful treatment would be anything that slows or stops progression of DM. “I would take anything that would slow down the progression of muscle wasting and let me lead a productive life,” said one man with DM1. “I’m not the person I was or want to be” [8]. One woman with DM2 suggested that treatments to improve breathing or stabilize the sleep-wake cycle might slow progression [8]. Improved treatments for pain were also mentioned by several people. The patients’ wish list for treatments also included those that reverse symptoms, although one man who mentioned this acknowledged the need to manage expectations.

## Conclusions

Slowing or stopping disease progression is viewed by people with DM as the most important objective of therapy, yet it remains unclear how or whether drug developers might assess disease slowing, in light of the substantial heterogeneity among patients, inter-individual variability over time, and gradual progression. In the absence of disease-modifying therapies, people with DM have relied primarily on off-label or OTC symptomatic treatments with varying degrees of success. Current drug development efforts target both symptom management and the underlying mutation, and both approaches have merit for those living with this disease. Next steps in understanding progression should include the further study of subpopulations to advance understanding of progression in this disease and efforts to drive patient enrichment strategies for upcoming clinical trials.
